# Perceived Patient-Provider Communication Quality and Sociodemographic Factors Associated With Watching Health-Related Videos on YouTube: A Cross-Sectional Analysis

**DOI:** 10.2196/13512

**Published:** 2019-05-17

**Authors:** Aisha Langford, Stacy Loeb

**Affiliations:** 1 Department of Population Health NYU Langone Health New York, NY United States; 2 Department of Urology and Population Health NYU Langone Health New York, NY United States

**Keywords:** social media, communication, health communication, ethnic groups, physician-patient relations, emotions, attention, cross-sectional studies, logistic models, HINTS

## Abstract

**Background:**

Approximately 73% of US adults use YouTube, making it the most popular social media platform. Misinformation on social media is a growing concern; recent studies show a high proportion of misinformative health-related videos. Several studies on patient-provider communication and general health information seeking have been conducted. However, few studies to date have examined the potential association between patient-provider communication and health information seeking on specific social media platforms such as YouTube. A better understanding of this relationship may inform future health communication interventions.

**Objective:**

The aim was to use nationally representative cross-sectional data to describe the association between perceived patient-provider communication quality and sociodemographic factors on watching YouTube health-related videos.

**Methods:**

Data from the 2018 Health Information National Trends Survey were analyzed (N=3504). The primary outcome was whether participants watched a health-related video on YouTube over the past 12 months. A patient-provider communication composite score was created by summing responses about how often providers did the following: (1) gave you the chance to ask all the health-related questions you had, (2) gave attention to your feelings, (3) involved you in health care decisions as much as you wanted, (4) made sure that you understood the things you needed to do to take care of your health, (5) explained things in a way that you could understand, (6) spent enough time with you, and (7) helped you deal with feelings of uncertainty. Sociodemographic factors included age, gender, race/ethnicity, and education. Descriptive statistics and multivariable logistic regression were conducted.

**Results:**

Approximately 1067 (35% weighted prevalence) participants reported watching a health-related video on YouTube. Higher perceived quality of patient-provider communication on the composite score was significantly associated with lower odds of watching health-related videos on YouTube. Regarding sociodemographic factors, increasing age and being a high school graduate (compared with college graduate) were associated with lower odds of watching health-related videos on YouTube; whereas, Hispanic and non-Hispanic Asians were more likely to have watched a health-related video on YouTube. For individual aspects of patient-physician communication, two of seven patient-provider communication variables were significant. Those who reported that providers “sometimes” spent enough time with them had higher odds of watching a health-related video on YouTube, compared with those who said providers “always” spent enough time with them. Participants reporting that they “never” have a chance to ask all their health-related questions also had higher odds of watching health-related videos on YouTube compared with those who reported “always.”

**Conclusions:**

Higher perceived quality of patient-provider communication is associated with lower odds of watching health-related videos on YouTube. When providers do not spend enough time or give an opportunity to ask questions, patients are more likely to pursue health information on social media.

## Introduction

### Background

Historically, patient-provider communication has been associated with various health outcomes [1-3] and health information-seeking behaviors [4,5]. For example, one study found that problems with patient-centered communication and clinical care coordination were associated with a higher likelihood of independent eHealth engagement [6], whereas another study found that internet health information seeking could improve the patient-physician relationship depending on the history of the relationship and whether the patient discussed the information with their doctor [4]. However, an underexplored area of research is the association between patient-provider communication and health information seeking on specific social media platforms. As of January 2018, the Pew Research Center reported that nearly 70% of Americans use at least one social media site [7]. By popularity, YouTube ranks first with 73% of Americans using the site, followed by Facebook at 69% and Instagram at 37% [8].

Given the popularity of social media and relative ease in which information can be posted online, health-related misinformation on social media has become a growing public health concern that may affect patient-provider communication [9-12]. A number of studies have explored how and why patients use social media for health-specific purposes [13-15]. For example, Benetoli et al [16] conducted focus groups with Australian consumers and found that blogs helped consumers learn about other people’s experiences with the same condition, Facebook allowed them to follow health-related pages of interest and participate in disease-specific group discussions, Wikipedia was used to help gather information about health conditions and treatments, and YouTube was used to learn about medical procedures including surgery. Other studies have evaluated both the quantity and quality of YouTube video content for various health conditions and behaviors including prostate cancer, infertility, and smoking [11,17-24], as well as, more broadly, how YouTube videos tags are assigned and described by the disseminator [25].

Although many health-related videos on YouTube are deemed educationally useful and are of high quality [22,26-29], some studies show that health-related videos on YouTube are often of poor quality, misleading, and/or have commercial content designed to sell products or services [11,12,30-32]—all which may have serious implications for consumer attitudes and medical decision making. For example, our group recently examined the top 150 YouTube videos on prostate cancer and found that 77% had misinformative and/or biased content in the video or comments, and that there was an inverse relationship between views and thumbs up with expert-rated quality [11]. Another study showed that YouTube videos portraying immunization negatively were more highly rated by users than positive videos, but 45% of the negative videos contained misinformation [33]. In general, viewer engagement with YouTube videos appears to be higher when health-related videos contain personal stories, misinformation, and/or nonrecommended therapies [11,17,34,35].

### Theory Considerations and Health Communication

The rapid growth of internet and social media use over the past 20 years also has implications for health communication or “the study and use of communication strategies to inform and influence individual and community decisions that enhance health” [36]. Health communication strategies may include, but are not limited to, the exchange of information between individuals, development of health messages, providing multiple ways for people to access health information, and ensuring that health information meets the needs of people at varying health literacy levels [37]. Although no single theory or model captures all the factors affecting health communication, theories can be combined to better understand these processes; this decision should be based on the health issue or problem, target population, and context [38]. For example, the Basic Communication Model highlights that source and channel, message, and audience are the key elements of communication [39]; whereas, the Chronic Care Model posits that an activated patient and a prepared, proactive health care team are needed to have productive interactions [40]. Street’s [41] Ecological Model of Communication in Medical Encounters helps explain “how communication in these interactions is (or can be) affected by the interpersonal, organizational, media, political-legal, and cultural environments within which they take place.” This model also suggests that patients and providers have predisposing factors such as communication style, verbal and nonverbal behaviors, and cognitive-affective influences that impact the patient-provider communication process [41]. The Technology Acceptance Model, which is an adaption of the Theory of Reasoned Action, was originally developed to assess perceived usefulness and ease of use of technology in workplace settings [42-44] but has also been used to understand consumer acceptance of health technology [45-47].

More broadly, information processing theories such as the Elaboration Likelihood Model can also be informative in the contexts of patient-provider communication and information seeking on social media [48,49]. The Elaboration Likelihood Model suggests that persuasive messages are processed through one of two routes: central or peripheral. Central route processing occurs when people are highly motivated to think about an issue (and have sufficient time and cognitive resources to do so), which should lead to greater “elaboration” or thoughtful, deliberate weighing of the message attributes. In contrast, peripheral route processing occurs in low motivation situations in which people are more prone to rely on peripheral cues such as a celebrity endorsement of a product or behavior, or the esthetic features of the health information product. Taken together, the aforementioned theories and the Health Information National Trends Survey (HINTS) framework [50] might suggest that patient-provider communication may affect how a patient’s health information needs are met, which in turn may influence their health information-seeking behaviors, potentially leading them to seek health information on social media platforms.

The purpose of this study was to describe the association between perceived patient-provider communication quality and sociodemographic factors on the likelihood of watching of health-related videos on YouTube. We hypothesized that younger people, minorities, and individuals who do not feel like they had enough time with their provider or did not have their questions answered would be more likely to seek health information on YouTube. This research is important because it may shed light on which specific patient-provider communication aspects are associated with health information seeking on social media and which patient subgroups are more likely to use YouTube to watch health-related videos, both of which may inform future health communication interventions. Social media platforms including YouTube have great potential for the delivery of behavioral interventions, but this area of research is still in its infancy. A better understanding of which health consumers are using these platforms for health information and what perceived gaps they fill could help with this burgeoning field.

## Methods

### Brief Overview of the Health Information National Trends Survey

**Figure 1:**
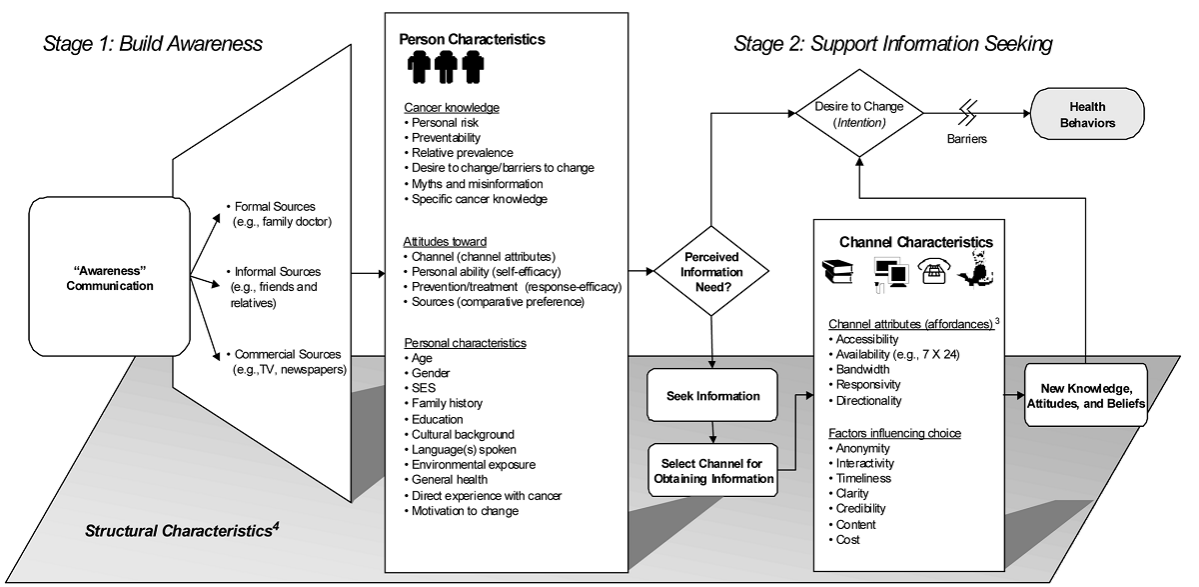
Health Information National Trends Survey (HINTS) framework, which relates psychosocial characteristics to the dynamic process of becoming aware of, and then seeking new information on, public health messages. Source: [50]; reprinted with permission from Taylor & Francis Ltd.

HINTS is a probability-based, nationally representative cross-sectional survey of noninstitutionalized US adults aged 18 years and older that was designed to monitor trends in the American public’s use of cancer and other health-related information. The survey was developed by the National Cancer Institute’s Health Communication and Informatics Research Branch and has been administered approximately every 2 years since 2003. HINTS is guided by a conceptual framework (see Figure 1) informed by the communication and behavioral science literature. To help ensure participant understanding, HINTS surveys involve at least two rounds of cognitive testing; field testing is also conducted. Criteria for inclusion of survey items are based on scientific validity (ie, use of established measures for assessing constructs of interest), utility of information for key stakeholders, and implementation considerations [50]. For race and ethnicity, blacks and Hispanics are oversampled to help enhance minority representation. Full details about general HINTS methodology are documented elsewhere [50-52].

This study evaluated participant data from HINTS 5, Cycle 2. The sample design consisted of two stages. In the first stage, an equal probability sample of addresses was selected from within each explicit sampling stratum. In the second stage, one adult was selected within each sampled household using the next birthday method. The sampling frame consisted of a database of addresses used by Marketing Systems Group to provide random samples of addresses. Data were collected between January and May 2018 via a mailed survey. A US $2 prepaid monetary incentive was included to encourage participation. Benefits of the sampling approach include enhanced geographic and demographic diversity. Additionally, data collection was anonymous and done via a pen-and-paper survey, two strategies to help reduce social desirability bias. Complete data for HINTS 5, Cycle 2, were collected from 3504 respondents and the overall survey response rate was 32.9%. As this study involved an analysis of a nonidentified publicly available data, institutional review board review approval was not required.

### Measures

#### Health-Related Video on YouTube

The primary outcome was whether participants watched a health-related video on YouTube over the past 12 months (yes/no).

#### Patient-Provider Communication

A patient-provider communication composite score was created by summing responses to questions about how often doctors, nurses, or other health care professionals did the following: (1) gave you the chance to ask all the health-related questions you had, (2) gave the attention needed for your feelings and emotions, (3) involved you in health care decisions as much as you wanted, (4) made sure that you understood the information needed to take care of your health, (5) explained things in a way that you could understand, (6) spent enough time with you, and (7) helped you deal with feelings of uncertainty. Per Epstein and Street [53], these survey items represent key aspects of patient-centered care and have been used in several studies [6,54-58]. Response options were recoded so that a higher number would indicate more positive patient-provider communication (ie, 1=never, 2=sometimes, 3=usually, and 4=always). The minimum and maximum patient-provider composite scores were 7 and 28, respectively. The Cronbach alpha for the seven patient-provider communication items was .92; this high internal consistency provides support for creating a composite score. Patient-provider communication items were also evaluated individually.

#### Sociodemographic Factors

Age, gender (male/female), race/ethnicity (white, black, Hispanic, Asian), and education (less than high school, high school graduate, some college, and college graduate) were evaluated.

### Statistical Analyses

Per the HINTS analytic recommendations, replicate weights were applied to compute accurate standard errors for statistical testing procedures and to estimate US population-level percentages. Descriptive and inferential statistics including chi-square and *t* tests, and two multivariate logistic regression models were conducted. The first logistic regression model explored the association between watching a health-related video on YouTube and the patient-provider composite score, age, gender, race/ethnicity, and education. The second logistic regression model explored the association between watching a health-related video on YouTube and the seven individual patient-provider communication items, age, race/ethnicity, and education. Gender was removed from the second model because it was not significant in either model. All analyses were conducted using Stata 14.2 [59]. We excluded missing data from the analyses.

## Results

As shown in Table 1, the participants included in this analysis were mostly non-Hispanic white, with some college, and a mean age of 48.9 (SE 0.3) years. The overall mean composite score for patient-provider communication was 23.8 (SE 0.1). Approximately 1067 participants (35% weighted prevalence) reported watching a health-related video on YouTube in the last 12 months. Participants who reported watching a health-related video on YouTube were generally younger (eg, 66% were younger than 50 years old) and slightly more were female (54%). Those who watched a health-related video on YouTube had a lower mean composite score for patient-provider communication compared to those who did not report watching a video (mean 23.1, SE 0.2 vs mean 24.2, SE 0.1; *t*_49_=−3.74, *P*<.001). Additionally, compared with those who did not report watching a health-related video on YouTube, those who watched a health-related video on YouTube had lower percentages for endorsing that health care providers “always” did the seven patient-provider communication behaviors listed (versus never, sometimes, or usually).

In the first multivariable logistic regression model (Table 2), a higher composite score for patient-provider communication (odds ratio [OR] 0.95, 95% confidence interval [CI] 0.92-0.99, *P*=.02) and increasing age (OR 0.96, 95% CI 0.95-0.97, *P*<.001) were associated with lower odds of having watched a health-related video on YouTube. Compared with non-Hispanic whites, Hispanics and non-Hispanic Asians had higher odds of watching a health-related video on YouTube (OR 1.65, 95% CI 1.13-2.42, *P*=.01 and OR 2.40, 95% CI 1.19-4.81, *P*=.02, respectively). Regarding education, high school graduates had lower odds of having watched a health-related video on YouTube compared with those with a college degree or higher (OR 0.57, 95% CI 0.39-0.85, *P*=.007).

In the second multivariable logistic regression model that evaluated the seven patient-provider communication items individually (Table 3), only two items were significant. Compared with those who said providers “always” spent enough time with them, those who reported that providers “sometimes” spent enough time with them had higher odds of watching a health-related video on YouTube (OR 1.92, 95% CI 1.17-3.14, *P*=.01). Additionally, participants who said providers “never” give them to chance to ask all their health-related questions had higher odds of watching a health-related video on YouTube compared with those who said providers “always” did so (OR 4.78, 95% CI 1.16-19.63, *P*=.03). Age, race/ethnicity, and education remained significant.

**Table 1:** Participant characteristics according to watching health-related videos on YouTube.

Characteristic and category (raw counts)		All (N=3504)	Watched YouTube video (n=1067)	Did not watch YouTube video (n=2361)	*P* value^a^
Age (n=3417), mean (SE)^a^		48.9 (0.3)	42.0 (0.8)	52.6 (0.6)	<.001
**Age group (years), weighted %^a^**					**<.001**
	18-34 (n=406)	24	36	16	
	35-49 (n=658)	27	30	25	
	50-64 (n=1113)	30	26	32	
	65-74 (n=736)	11	6	14	
	≥75 (n=504)	8	2	11	
**Gender, weighted %^a^**					**.11**
	Men (n=1394)	49	46	50	
	Women (n=2054)	51	54	49	
**Race/Ethnicity, weighted %^a^**					**<.001**
	Non-Hispanic white (n=1983)	67	59	71	
	Non-Hispanic black (n=444)	11	11	11	
	Hispanic (n=461)	17	20	15	
	Non-Hispanic Asian (n=138)	5	9	3	
**Education, weighted %^a^**					**<.001**
	Less than high school (n=275)	9	6	10	
	High school graduate (n=631)	22	16	26	
	Some college (n=1039)	40	45	38	
	College degree (n=1508)	29	34	26	
Patient-Provider communication composite score (n=2871), mean (SE)^a^		23.8 (0.1)	23.1 (0.2)	24.2 (0.1)	<.001
**Patient-Provider communication individual items,^b^** **weighted %**					
	Chance to ask health-related questions (n=2945)	63	57	65	.001
	Attention needed for your feelings and emotions (n=2936)	49	43	51	.05
	Involved you in health care decisions (n=2933)	57	51	59	.007
	Made sure you understood things needed to do (n=2936)	65	62	68	.07
	Explained things in a way that you could understand (n=2933)	66	61	69	.12
	Spent enough time with you (n=2923)	48	40	53	<.001
	Helped you deal with feelings of uncertainty (n=2917)	47	41	50	.06

^a^Testing for differences in distributions between those who have and have not watched a health-related video on YouTube.

^b^Values for these variables represent the percent of people who answered “always.”

**Table 2:** Multivariable logistic regression of the association between patient-provider communication composite score, sociodemographic factors, and watching health-related videos on YouTube (N=2408).

Item		OR^a^ (95% CI)	*P* value
Patient-Provider communication score (continuous)		0.95 (0.92-0.99)	.02
Age (continuous)		0.96 (0.95-0.97)	<.001
**Gender**			
	Men (ref^b^)	1.0	
	Women	1.25 (0.91-1.71)	.15
**Race/Ethnicity**			
	Non-Hispanic white (ref)	1.0	
	Non-Hispanic black	1.25 (0.80-1.96)	.31
	Hispanic	1.65 (1.13-2.42)	.01
	Non-Hispanic Asian	2.40 (1.19-4.81)	.02
**Education**			
	≥College degree (ref)	1.0	
	Some college	1.14 (0.82-1.58)	.41
	High school graduate	0.57 (0.39-0.85)	.007
	Less than high school	0.66 (0.29-1.47)	.31

^a^OR: odds ratio.

^b^ref: reference.

**Table 3:** Multivariable logistic regression of the association between individual patient-provider communication items, sociodemographic factors, and watching health-related videos on YouTube (N=2427).

Item		OR^a^ (95% CI)	*P* value
**Chance to ask health-related questions**			
	Always (ref^b^)	1.0	
	Usually	1.06 (0.72-1.54)	.75
	Sometimes	0.49 (0.22-1.08)	.08
	Never	4.78 (1.16-19.63)	.03
**Attention needed for your feelings and emotions**			
	Always (ref)	1.0	
	Usually	0.85 (0.53-1.36)	.49
	Sometimes	0.95 (0.50-1.79)	.88
	Never	0.81 (0.31-2.13)	.67
**Involved you in health care decisions**			
	Always (ref)	1.0	
	Usually	1.11 (0.70-1.74)	.64
	Sometimes	1.50 (0.78-2.88)	.21
	Never	3.17 (0.83-12.08)	.09
**Made sure you understood the things you needed to do**			
	Always (ref)	1.0	
	Usually	0.97 (0.60-1.58)	.93
	Sometimes	0.98 (0.46-2.11)	.97
	Never	0.72 (0.19-2.75)	.63
**Explained things in a way that you could understand**			
	Always (ref)	1.0	
	Usually	0.94 (0.51-1.73)	.84
	Sometimes	0.73 (0.26-2.04)	.55
	Never	0.45 (0.06-3.29)	.43
**Spent enough time with you**			
	Always (ref)	1.0	
	Usually	1.39 (0.90-2.13)	.12
	Sometimes	1.92 (1.17-3.14)	.01
	Never	2.62 (0.83-8.22)	.10
**Helped you deal with feelings of uncertainty**			
	Always (ref)	1.0	
	Usually	0.89 (0.52-1.51)	.68
	Sometimes	0.96 (0.48-1.92)	.93
	Never	0.63 (0.26-1.53)	.31
Age (continuous)		0.96 (0.95-0.97)	<.001
**Race/Ethnicity**			
	Non-Hispanic white (ref)	1.0	
	Non-Hispanic black	1.29 (0.82-2.02)	.26
	Hispanic	1.68 (1.12-2.53)	.01
	Non-Hispanic Asian	2.27 (1.12-4.60)	.02
**Education**			
	College degree (ref)	1.0	
	Some college	1.14 (0.82-1.58)	.42
	High school graduate	0.57 (0.38-0.85)	.007
	Less than high school	0.66 (0.32-1.39)	.28

^a^OR: odds ratio.

^b^ref: reference.

## Discussion

### Principal Findings

The purpose of this study was to describe the association between perceived patient-provider communication quality and sociodemographic factors on watching health-related videos on YouTube. In summary, we found that perceived patient-provider communication quality (measured on a composite scale and as individual items), age, race/ethnicity, and education were significantly associated with watching health-related videos on YouTube. Our findings are in line with previous studies showing poor patient-provider communication was associated with higher online health information seeking [6,60] and sociodemographic differences in use of social media platforms [8,61-63]. New findings are that higher perceived patient-provider communication quality is associated with lower odds of watching health-related videos on YouTube.

To date, several studies have used HINTS data to explore different aspects of patient-provider communication including disparities in communication [55], the effects of health utilization and sociodemographic factors on patient-provider communication [64], degree of patient-centeredness in communication with cancer survivors [54], and the role of patient-centered communication on different types of eHealth usage [6]. Other studies have explored online health information seeking after medical visits broadly [60,65] and health care information on YouTube specifically [12]. However, ours is the first-known study to examine patient-provider communication in the specific context of watching health-related videos on YouTube. This research is important because there may be unique reasons that some people seek out health information on YouTube as compared with other channels. There may also be opportunities for health care providers to assist patients in finding credible sources of information on YouTube before, during, and after a medical visit. In addition, these data are important for further efforts to use YouTube for delivery of behavioral interventions, which is a burgeoning area of research.

### Patient-Provider Communication and Watching Health-Related Videos on YouTube

**Figure 2:**
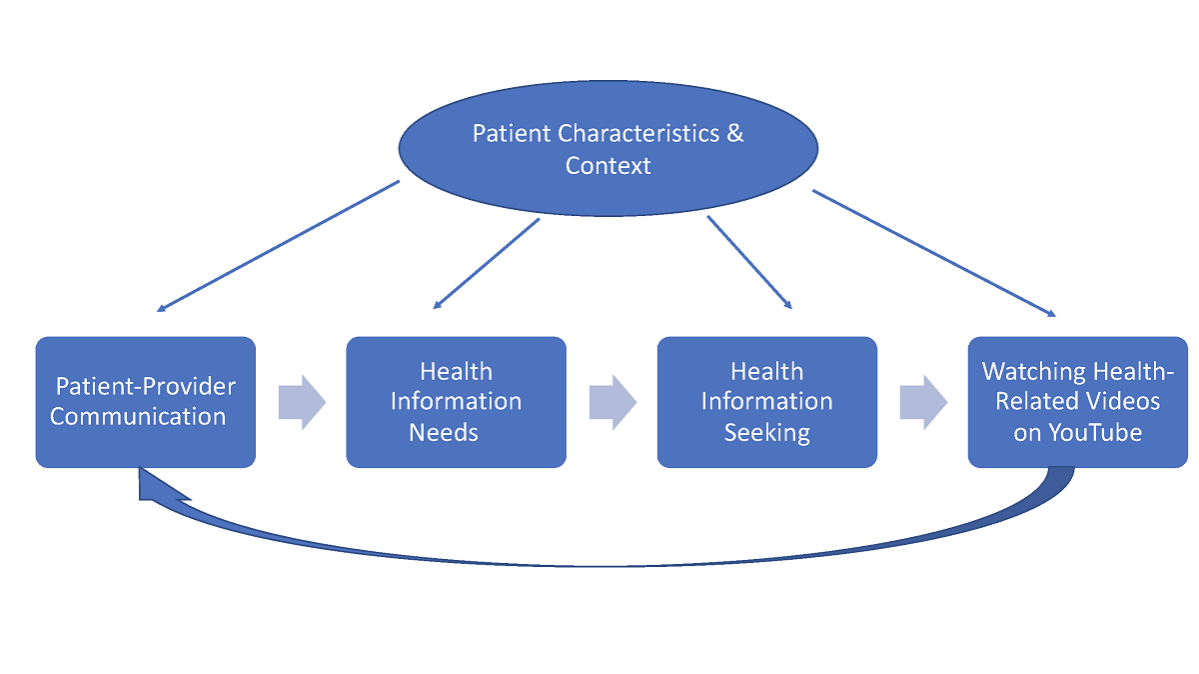
Conceptualization of the relationship between patient-provider communication quality and watching health-related videos on YouTube.

Overall, those with a higher perceived quality of patient-provider communication had lower odds of watching health-related videos on YouTube. Regarding the seven patient-provider communication items that were evaluated individually, only two were associated with watching health-related videos on YouTube: (1) “never” having a chance to ask all health-related questions and (2) “sometimes” feeling that health care provider spends enough time with you compared with people who reported that providers “always” did these behaviors. Although several studies have been conducted on the link between patient-provider communication and health outcomes, our findings provide a new window into the specific associations between patient-provider communication and health information seeking on YouTube. Our findings may support the idea of a feedback loop regarding patient-provider communication and health information seeking on YouTube (see Figure 2).

Poor patient-provider communication during a medical visit may have implications for how, if at all, a person’s health information needs are addressed. This can lead a person to conduct more health information seeking after the medical visit by watching health videos on YouTube because they still have questions or because they are anxious, which may potentially expose the patient to misinformation on YouTube. In turn, exposure to misinformation on YouTube may affect the quality of patient-provider communication in future medical visits because the time is largely spent discussing non-evidenced-based recommendations found on YouTube (eg, injecting herbs into the prostate will cure prostate cancer, immunization is dangerous) [11,33,66]. Consequently, there may be less time to talk about other issues, such as which pharmacological and nonpharmacological treatments may be most efficacious for the patient and the harms, benefits, and quality of life considerations associated with each option. Conversely, watching health-related videos on YouTube before or after a medical encounter may improve a patient’s knowledge of a health condition and related treatment options, which may enhance patient-provider communication in future visits because the patient is more informed and potentially clearer about their goals and preferences [4,67]. In both scenarios, patient characteristics and context will impact the patient-provider communication process, health information needs, and health information-seeking behaviors.

One can also argue that discussing *any* information that a patient finds online, regardless of a subjective assessment of whether that information is misinformative, provides additional opportunities to build the patient-provider relationship. For example, a hypertensive patient may seek out YouTube videos on “natural ways” to cure hypertension because they are concerned about overtreatment and potentially falling if their blood pressure is treated too aggressively. Another patient may be drawn to watching certain health-related videos on YouTube because of their illness beliefs [68], philosophies about medical interventions [69], or from a desire to learn more about how other patients such as them manage their condition [70]. If providers are nonjudgmental and invite discussion about *why* certain types of health information resonate with a patient, this may provide an opportunity for the provider to better understand the patient’s concerns, values, and preferences.

### Sociodemographic Factors and Watching Health-Related Videos on YouTube

We found that Hispanic and non-Hispanic Asian participants had higher odds of watching health-related videos on YouTube compared with white participants. Although we cannot fully explain this finding, a potential explanation may be due to cultural perspectives about the authority of health care professionals, which may lead these groups to be less likely to ask questions during medical visits, potentially leaving unanswered questions and leading them to seek out health videos on YouTube. Trust of health care professionals may have also played a role, which may have affected patient-provider communication. In a prior study using HINTS data from 2014, Singh et al [64] found that Hispanics and Asians reported lower quality patient-centered provider communication compared to whites; whereas, in a separate study on shared decision making, Levine et al [71] found lower scores for patient-perceived shared decision making in Asians compared to white adults.

We also found that as age increased, the odds of watching a health-related video on YouTube decreased. These findings are consistent with general trends in social media consumption by age [8,61,63]. Finally, regarding education, people who were high school graduates were 43% less likely to report watching a health-related video on YouTube compared to those with a college degree. It is likely that those with greater levels of education have greater self-efficacy for finding information online or are possibly exploring alternative knowledge sources beyond their health care providers. Moreover, lower levels of health technology acceptance [72,73] and eHealth literacy [74,75] among high school graduates may also partially explain this finding.

### Strengths, Limitations, and Future Directions

Our findings provide an important and nuanced contribution to the literature given that we evaluated two aspects of health communication typically examined separately: patient-provider communication and mass communication via social media. Strengths of this study include the use of HINTS, a large nationally representative survey designed to track changes in health communication and information technology in the United States. The questions asked in HINTS allowed us to examine perceived patient-provider communication quality and health information seeking on YouTube which is the most commonly used social media platform in the United States. Despite its strengths, some limitations should be noted. First, we do not know the reasons why participants were watching health-related videos on YouTube (eg, to learn about a screening test or disease), for whom they were watching (eg, themselves, parent, significant other), or the quality of the videos viewed. Second, the YouTube-related question in HINTS was general and did not provide a specific definition of what constituted a “health-related” video; therefore, we do not know what participants considered as being “health related” (eg, videos about getting a mammography, weight lifting, mindfulness meditation). HINTS 5, Cycle 2, did not include measures of eHealth literacy or the “ability to seek, find, understand, and appraise health information from electronic sources and apply the knowledge gained to addressing or solving a health problem” [76]. This may have affected how and why people sought out health information on YouTube.

### Directions for Future Research

This study raises several potential questions for future inquiry. First, it should be acknowledged that social media has the potential to support patients in several important ways, including meeting their informational and emotional needs [67]. Social media also has the potential to affect attitudes and beliefs in ways that are counter to the goals of many health care professionals (eg, people avoiding vaccines because of antivaxer messages on social media) [12,33]. A better understanding of why and how patients are using different social media platforms for health-related purposes may shed light on which needs that are not being addressed in the routine clinical encounters and how these needs may be partially supported with population-level approaches to health communication. Second, future work should explore the degree to which health care professionals are helping patients find health information on YouTube and which sources of information they are recommending. Third, future research should explore whether strategies to enhance patient-provider communication and access to interprofessional health care teams (eg, doctors, nurses, pharmacists, health coaches, consumer health librarians) affects the likelihood that patients will watch health-related videos on YouTube as team-based care theoretically provides multiple “touch points” and, thus, more opportunities for quality patient-provider communication. Fourth, as patients navigate the vast sea of health information on YouTube, further investigation is needed to understand the process by which patients determine whether health information is useful and credible.

Fifth, the notion of misinformation on social media has garnered a lot of attention in recent years [9,11,32]; however, it is not clear what exactly constitutes misinformation and who gets to determine whether something is misinformative. This raises other questions for health communicators regarding what the “gold standard” of health information should be when equipoise exists or there is disagreement between professional societies about guidelines. Relatedly, different studies have used different approaches to assess the quality of health information. Future work should aim to develop more standardized approaches to assessing and labeling content as misinformative. Sixth, further studies are needed on the impact of illness beliefs and representation on patient-provider communication and subsequent information seeking on YouTube [77-79]. It is possible that people who are watching health-related videos on YouTube are doing so because they (1) want to confirm the information given by their physicians, (2) simply want more information to better understand their health condition, or (3) disagree with the treatment plan recommended by their doctor and are exploring other options and knowledge sources that support their beliefs. The latter option supports the notion of “confirmation bias” or the concept that people seek out information that supports their preexisting beliefs [80,81]. Finally, further evaluation of which theory (or combination of theories and constructs) best explains the relationship between patient-provider communication and watching health-related videos on YouTube is needed. Notably, several concepts from various disciplines are relevant, including technology acceptance, eHealth literacy, information processing, social media self-efficacy, interpersonal communication, mass communication, and message framing.

### Conclusions

Lower perceived quality of patient-provider communication is associated with higher odds of watching health-related videos on YouTube. This study could have implications for health professionals (eg, try to give their patients enough time to ask all their questions) and for researchers seeking to use social media for health promotions to understand their potential audience. As social media grows in popularity, more research is needed on the relationship between the impact of patient-provider communication and health-related information seeking on YouTube. In particular, which features of health-related videos are most likely to engage consumers also needs to be examined so that health communication professionals can design population-level health communication interventions that are credible and evidence-based, yet still appealing to the audiences they are trying to reach.

## Abbreviations

HINTS: Health Information National Trends Survey
OR: odds ratio
